# Comparing Visual Self‐Assessment and Virtual Reality Training for Improving Basic Life Support Competency in Nursing Students: An Educational Study

**DOI:** 10.1002/hsr2.73202

**Published:** 2026-09-07

**Authors:** Mohsen Hamidian, Hamid Salehiniya, Anis Jor, Hakimeh Sabeghi

**Affiliations:** ^1^ Department of Nursing, School of Nursing and Midwifery Birjand University of Medical Sciences Birjand Iran; ^2^ Student Research Committee, School of Nursing and Midwifery Zahedan University of Medical Sciences Zahedan Iran; ^3^ Department of Epidemiology and Biostatistics, School of Health, Social Determinants of Health Research Center Birjand University of Medical Sciences Birjand Iran; ^4^ Department of Nursing, Faculty of Nursing and Midwifery Zabol University of Medical Sciences Zabol Iran; ^5^ Faculty of Nursing and Midwifery, Geriatric Health Research Center Birjand University of Medical Sciences Birjand Iran

**Keywords:** basic life support, competency, nursing students, virtual reality, visual self‐assessment

## Abstract

**Background and Aims:**

Cardiac arrest (CA) is an important medical emergency and a leading cause of death worldwide. Cardiopulmonary resuscitation (CPR) is an essential basic life support (BLS) and the initial response to CA. The success of resuscitation depends on the skills and performance of rescuers, as well as effective training to enhance knowledge and skills in this area. This study aimed to compare the effects of visual self‐assessment (VSA) and virtual reality (VR) training on nursing students' CPR competency.

**Methods:**

This educational study included 54 students who were randomly assigned to one of two groups of VSA and VR and received BLS training. A four‐section questionnaire assessed the students' demographic information, knowledge, attitude, and performance related to BLS before the intervention, immediately, and 2 months after the intervention. SPSS 27 and Mann–Whitney *U* test, independent samples *t*‐test, Friedman test, one‐way repeated measures ANOVA, and Wilcoxon post‐hoc test were employed for data analyses at a significance level < 0.05 based on normality test results.

**Results:**

Mean age of participants was 22.61 ± 1.64 years; 32 (59.3%) were female, and 23 (42.6%) were in 5 academic semester. Only 2 months after intervention, the median BLS knowledge score in VSA group was significantly higher than VR group (15 (14–15) vs. 14 (13.75–14)) (*p* < 0.001). The mean score of BLS attitude significantly increased to 21.03 ± 1.74 (*p* = 0.02) in the VSA group and to 20.40 ± 1.78 (*p* = 0.03) in the VR group. However, the differences between the two groups were not statistically significant before (*p* = 0.442), immediately after (*p* = 0.079), and 2 months after intervention (*p* = 0.195). Only at 2 months after intervention, the median BLS performance score in the VSA group was significantly higher than in the VR group (10 (9–10) vs. 9 (9–9)) (*p* < 0.001).

**Conclusion:**

Based on the study results, both VSA and VR training were effective in improving the nursing student's knowledge, attitude, and performance of BLS. However, VSA was partially more effective.

## 1 Introduction

Cardiac arrest is the sudden stop of heart activity, leading to unresponsiveness, lack of normal breathing, and no circulation. If corrective measures are not taken rapidly, this condition progresses to sudden death [1]. There are significant differences in survival rates after cardiac arrest (CA), which are not solely determined by the patient's condition. CPR guidelines emphasize the importance of timely and effective interventions, early defibrillation, high‐quality resuscitation (including minimizing interruptions in chest compressions), and addressing the underlying cause of CA to improve survival and outcomes [2, 3].

In hospital settings, nursing teams are generally the first to identify CA situations and initiate resuscitation efforts [2, 4]; therefore, the preparedness of nurses for high‐quality CPR improves patient survival chances. Proper CPR, performed based on guidelines, is essential for optimizing patient survival [5]. Evidence widely suggests that Basic Life Support (BLS) is the foundation of resuscitation efforts, making it a crucial skill for nurses before graduation [6]. BLS comprises a series of immediate interventions to preserve brain function until advanced medical help arrives. The primary goal of BLS is to restore partial blood flow to the brain and heart through high‐quality CPR and to correct a life‐threatening arrhythmia with early defibrillation [7]. BLS is periodically updated by international bodies such as the American Heart Association (AHA) and the International Liaison Committee on Resuscitation (ILCOR) based on extensive scientific evidence [7]. BLS training is essential for nursing students as they may encounter emergencies during their clinical practice. Additionally, nursing students should learn the latest and most appropriate knowledge on resuscitation and apply this theoretical knowledge in practice [5, 6].

While BLS competency is crucial, studies reveal that nurses and students often struggle to perform CPR based on international guidelines [2, 4, 6, 8, 9]. We can prevent the negative outcomes of poor BLS and CPR through effective education [10]. Therefore, effective training strategies are essential to overcome the limitations imposed on nurses and students as future professionals.

In 2020, the AHA guidelines recommended BLS training using methods such as simulations, high‐fidelity manikins, various feedback devices, and online courses [9, 11]. Various evidence suggests that clinical simulation is an essential part of nursing education [12, 13]. High‐fidelity simulation is widely used in nursing education, as it is ideal for practical and team‐based training where tactile feedback and real‐world interaction are crucial [10]. Based on Kolb's Experiential Learning Theory, learning is a process whereby knowledge is created through the transformation of experience. This theory supports the use of immersive technologies that allow learners to engage in concrete experiences (simulated CPR) followed by reflective observation and active experimentation [14]. While integrative reviews indicate that no single “best” method exists for BLS training, they highlight that self‐directed learning and immediate feedback significantly enhance student performance [6, 15]. Furthermore, international resuscitation guidelines suggest incorporating technology into CPR training as alternatives to standard training [16]. These interventions are facilitated by digital technologies such as instructional videos, web‐based learning, computer programs, mobile applications, advanced software, and VR [10].

However, a significant research gap remains regarding the comparative efficacy of low‐cost, accessible technological interventions versus traditional assessment methods. Specifically, while Virtual Reality (VR) offers immersive, high‐fidelity environments, its cost and accessibility can be limiting. Conversely, Visual Self‐Assessment (VSA)—defined as the process where learners record their CPR performance using standard video devices and self‐evaluate against checklist criteria using visual feedback—offers a feasible, low‐cost alternative that promotes reflective practice [10]. It is crucial to distinguish between these two approaches. VR training utilizes computer‐generated simulations to create an immersive 3D environment where users interact with virtual patients and equipment, providing immediate, automated feedback on compression depth, rate, and recoil without human intervention [17]. In contrast, visual self‐assessment relies on standard video recording and subsequent self‐evaluation against a standardized checklist, fostering metacognitive skills and self‐regulated learning but lacking the immersive interactivity of VR [18].

Based on the reviewed evidence, we hypothesized that integrating virtual reality into CPR training would provide a practical, realistic, and valuable clinical learning opportunity. Therefore, by operationalizing “BLS competency,” we tried to define it as the ability to perform CPR steps accurately, measured by global CPR performance scores, compression depth, compression rate, and hands‐free intervals, as assessed by a validated standardized checklist and/or performance monitoring device. Aligning with the goal of nursing education programs to ensure high‐quality CPR competency, we aimed to compare the effects of visual self‐assessment and VR on BLS training in nursing students.

## 2 Methods

### 2.1 Study Design and Setting

This educational study was conducted in 2024 (October 22, 2024 to December 22, 2024). The study population consisted of nursing students at Zabol University of Medical Sciences. Second‐ and third‐year undergraduate nursing students who had not participated in any formal training, workshop, or class related to BLS or CPR prior to the initiation of the study, and who had no physical conditions that would impede the performance of CPR, were included in the study upon providing informed consent. Participants with irregular participation in the training workshop and incomplete questionnaires were excluded.

### 2.2 Allocation to Groups and Randomization

Based on the findings of Zahedmehr et al. [19], a sample size of 27 participants per group was calculated, considering the reported means (16.30 and 18.20) and standard deviations (2.46 and 3.02) in the two groups, 80% power, and 90% confidence level. Consequently, 54 third‐ and fourth‐year nursing students were randomly assigned to either the VSA or the VR group.

To balance the number of samples allocated to each of the study groups, the eligible participants were divided into two groups in the randomized block sampling method. According to the sample size, by using Excel software, 13 blocks of 4 and one block of 2 were created with different and random combinations in terms of the order of letters A and B. Blocks of 4 included two letters A and two letters B, and blocks of two included one of these two letters. Letter A corresponds to the VR group, and letter B corresponds to the VSA group. Then, at each stage, based on the table of random numbers, a block was randomly selected, and based on it, participants were assigned to one of the two study groups. This process continued until the sample size was completed.

Due to the nature of the interventions (video‐based vs. virtual reality), it was not possible to blind the participants and instructors. However, to minimize bias, the outcome assessor was not involved in the instruction and was blinded to the group allocation of the participants, and the statistical analyst remained blinded to the group assignments during data analysis.

### 2.3 Data Collection Tools

Data collection was done using a 4‐part researcher‐made questionnaire including demographic information (gender and academic semester), BLS knowledge, attitude, and performance questionnaire. The knowledge assessed by 15 four‐choice questions, which were scored 1for true answer and 0 for false and total score ranged from 0 to 15. In this section, we asked about principles of CPR such as “chest compression/ventilation ratio”, “correct place to check an adult's pulse”, “The best place for a heart massage”,

Students' attitudes towards BLS were assessed through 8 four‐choice questions. Scoring was on a Likert scale (strongly disagree (1), disagree (2), agree (3), and strongly agree (4). Total score ranged from 8 to 32. In this part, we assessed the students' attitude toward the essentials of learning theoretical and practical performance of BLS and CPR during the study period.

Practical score of students was assessed through an observational 10‐question scale after one‐person performance of BLS in a designed scenario for a hypothetical electrocution victim on a half‐body CPR torso mannequin. A score of 1 was considered for correct performance of the skill, and a score of zero was considered otherwise. The 10 investigated items were “scene and victim assessment”, “airway opening”, “breathing check”, “pulse check (if taught)”, “hand position for compressions”, “compression quality”, “compression rate and ratio”, “ventilation technique”, “minimizing interruptions”, and “post‑resuscitation and recovery”. Based on this questionnaire, a higher score indicated higher knowledge, attitude, and performance.

The validity and reliability of the BLS knowledge, attitude, and performance questionnaire were assessed before data collection. Content validity was established through both qualitative and quantitative methods. Qualitatively, the initial draft was reviewed by 10 experts (including physicians, emergency managers, and senior EMTs) who provided feedback on grammar, clarity, item placement, and alignment with study objectives. Quantitatively, content validity was calculated using the Content Validity Ratio (CVR) and Content Validity Index (CVI). For CVR calculation based on Lawshe's formula [20], experts rated each item as “essential”, “useful but not essential”, or “not essential”. With 10 experts, the critical CVR value was set at 0.62; items scoring above this threshold were retained, yielding an overall mean CVR of 0.75. The CVI was computed using Walters and Basch's method, where experts rated items on relevance, clarity, and simplicity using a 4‐point Likert scale [21]. Items with a CVI greater than 0.78 were included in the final version. The reliability of the questionnaires was evaluated using Cronbach's alpha, yielding a coefficient of 0.74 based on a pilot test with 20 students (not part of the main sample). The inter‐rater reliability of the performance checklist was found to be 89%.

### 2.4 Intervention

In this study, participants were divided into two groups: the VR group and the Video‐self‐assessment (VSA) group. Before the intervention, all participants from both groups completed a knowledge and attitude questionnaire and performed a BLS procedure for 3 min on a torso manikin. This served as a pre‐test within a simulated electrocution scenario.

Following the pre‐test, both groups attended a 2‐h theoretical training session on BLS administration aligned with the AHA (2020) guidelines, led by a Master of Science student in Emergency Nursing. After the theoretical instruction, the practical training phase began. Participants in the VR group practiced the BLS procedure for 3 to 5 min using a CPR training simulator approved by ARAS Company in Tabriz. The simulator uses VR, including intelligent positioning, simulated environments, body tracking, handheld controllers, headsets, and an AI coach. The angle of the compressions and the user's movements were transmitted to a main processor through specialized goggles, allowing the simulator AI coach to provide real‐time visual feedback on the correctness of the techniques used via a monitor. Until the participant performed a step correctly, the virtual trainer would not allow him to perform the next step. After that, participants received short feedback from a human coach (Master of Science in Emergency Nursing) about their performance.

Participants in the VSA group conducted their practical training for 3–5 min on a standard torso manikin. Students recorded their performance with personal mobile phones. Subsequently, students individually reviewed and critically analyzed their recorded videos to identify their strengths and weaknesses. After that, the Master of Science in Emergency Nursing provided short feedback to each participant regarding the correct or incorrect execution of the procedures.

In addition, participants in both groups watched their peers' performance. Immediate post‐training and 2‐month follow‐up assessments were conducted. During each post‐test, participants once again completed the knowledge and attitude questionnaire and performed the BLS procedure under the same conditions as the pre‐test, in the presence of an evaluator. The practical skills of the students during each test were evaluated by a Master of Science student in Emergency Nursing who was not involved in the instruction and was blinded to the group allocation of the participants. The knowledge and attitude questionnaires were completed by the students through self‐reporting and under direct supervision by the research team in a controlled classroom environment. Students were seated apart from one another to prevent unauthorized collaboration, and they were explicitly instructed to complete the tests independently without consulting external resources or peers. Proctors remained present throughout the testing period to ensure adherence to these protocols.

### 2.5 Statistical Analysis

The data were analyzed using SPSS 27. Data were described by frequency, frequency percent, mean, and standard deviation (SD). Data normality was assessed using the Kolmogorov–Smirnov test. Mann–Whitney *U* test or independent samples *t*‐test, Friedman test or one‐way repeated measures ANOVA, and Wilcoxon post‐hoc test were employed for data analyses at a significance level < 0.05 based on normality test results.

### 2.6 Ethics Approval and Consent to Participate

The current study was conducted in accordance with the Declaration of Helsinki. The Ethics Committee of Birjand University of Medical Sciences approved this study with the code of ethics IR. BUMS.REC.1403.191. Informed consent to participate was obtained from all the participants, and an assurance was provided regarding the confidentiality of their data. This study was a simulation‐based educational intervention involving nursing students and, according to applicable institutional/national requirements, was not subject to prospective clinical trial registration.

## 3 Results

**Figure 1:**
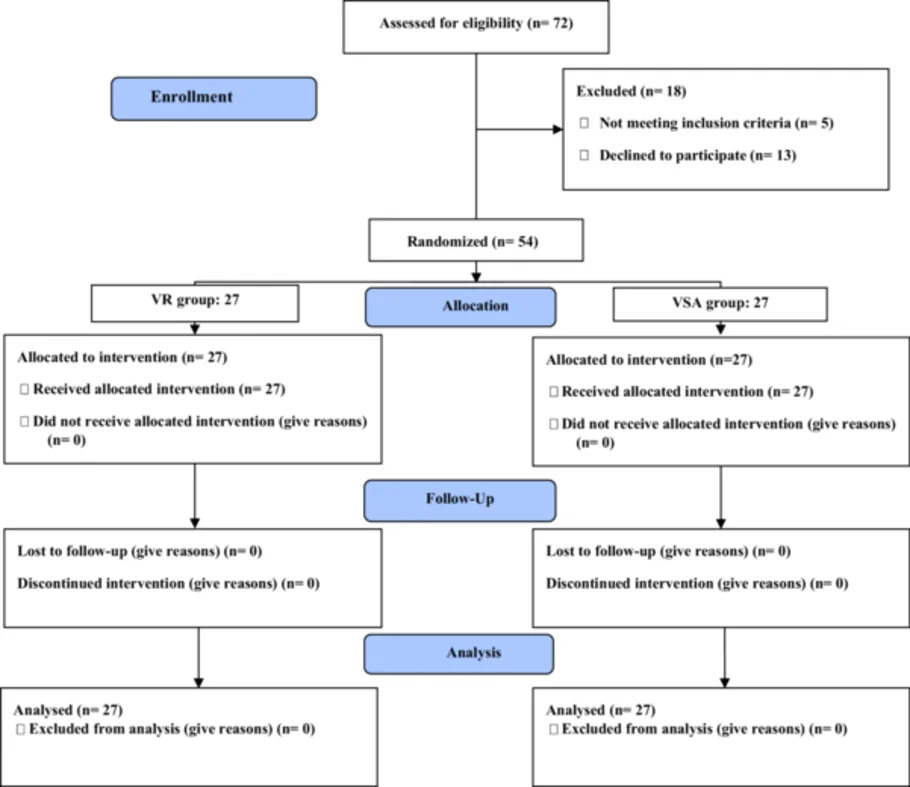
Flow diagram.

In this study, 54 students participated without attrition (Figure 1). The mean age of participants was 22.61 ± 1.64 years; 32 (59.3%) were female, and 23 (42.6%) were in 5 academic semester. More details based on study groups are presented in Table 1.

**Table 1:** Comparison of demographic variables between VSA and VR groups.

Variable/group	VSA	VR
Age (mean ± SD)	23.30 ± 1.86	21.93 ± 1.04
Gender (*N* (%))		
Male	10 (37)	12 (44.4)
Female	17 (63)	15 (55.6)
Academic semester (*N* (%))
3	13 (48.1)	10 (37)
4	4 (14.8)	9 (33.3)
5	7 (25.9)	4 (14.8)
6	(11.1)	4 (14.8)

Before intervention, the mean BLS knowledge score in the VSA group was 8.33 ± 1.83, which was not significantly different from the VR group (*p* = 0.1). Based on the Friedman test, BLS knowledge score significantly increased in both groups over time (*p* < 0.001), indicating an improvement following the intervention. The median score of BLS knowledge immediately after the intervention was not significantly different between the two groups (14 (13–14) vs. 14 (13–14.25), *p* = 0.729). But 2 months after the intervention, the median score of BLS knowledge was significantly higher in the VSA group (15 (14–15) compared to the VA group (14 (13.75–14)) (*p *< 0.001). Wilcoxon post‐hoc test indicated significant increases in knowledge scores of the VSA group immediately and 2 months later compared to before the intervention. In the VR group, significant increases in knowledge scores were also observed immediately and 2 months later compared to before the intervention (Table 2).

**Table 2:** Comparison of mean knowledge scores between the VSA and VR groups before, immediately, and 2 months after the intervention.

Groups time	VSA	VR	*p* value (between‐groups)
Before the intervention			
(Mean ± SD)	8.33 ± 1.83	9.23 ± 2.25	0.100^a^
95% CI for mean	7.60–9.06	8.32–10.14	
Immediately after the intervention (median (Q1–Q3))	14 (13–14)	14 (13–14.25)	0.729^b^
Two months after the intervention (median (Q1–Q3))	15 (14–15)	14 (13.75–14)	< 0.001^b^
Effect Size (Kendall's W) *p* value (within‐groups)	*W* = 0.872 < 0.001^c^	*W* = 0.832 < 0.001^c^	

^a^Independent samples *t*‐test.

^b^Mann–Whitney *U* test.

^c^Friedman test.

The mean score of BLS attitude in the VSA group significantly increased from 19.33 ± 1.83 before intervention to 20.77 ± 1.73 and 21.03 ± 1.74 immediately and 2 months after the intervention (*p *= 0.02). Also, this score in the VR group, significantly increased from 19.41 ± 2.16 before intervention to 19.85 ± 2.05 and 20.40 ± 1.78 immediately and 2 months after the intervention (*p *= 0.03), (Table 3). The result of one‐way repeated measures ANOVA test showed that this increase occurred due to the Time effect (*p *< 0.0001) (Table 4). However, the results showed that the mean score of BLS attitude was not significantly different before (*p *= 0.442), immediately after (*p *= 0.0.79), and 2 months after (*p *= 0.195) intervention between the two study groups (Table 3).

**Table 3:** Comparison of mean attitude scores between the VSA and the VR groups before, immediately, and 2 months after the intervention.

Attitude score	VSA	VR	*p* value (between‐groups)
Before the intervention			
Mean ± SD	19.63 ± 2.40	19.15 ± 2.16	0.442^a^
95% CI for mean	18.68–20.58	18.29–20.00	
Immediately after the intervention			
Mean ± SD	20.78 ± 1.73	19.85 ± 2.05	0.079^a^
95% CI for mean	20.09–21.46	19.04–20.66	
Two months after the intervention			
Mean ± SD	21.03 ± 1.74	20.41 ± 1.78	0.195^a^
95% CI for mean	20.35–21.73	19.70–21.11	
*p* value (within‐groups)	0.02^b^	0.03^b^	

^a^Independent *t*‐test.

^b^Repeated measures ANOVA.

**Table 4:** Results of one‐way repeated measures ANOVA.

Changes	*F*	*p* value	Partial eta squared
Group‐by‐time interaction effect	0.23	0.791	0.005
Group effect	3.07	0.085	0.142
Time effect	8.58	< 0.0001	0.056

Before intervention, the mean BLS performance score in the VSA group was 4.18 ± 1.30, which was not significantly different from the VR group (*p* = 0.467). Based on the Friedman test, BLS performance score significantly increased in both groups over time (*p* < 0.001), indicating an improvement following the intervention. The median score of BLS performance immediately after the intervention was not significantly different between the two groups (9 (9–9) vs. 9 (8–10), *p* = 0.780). But, 2 months after the intervention the median score of BLS performance was significantly higher in VSA group (10 (9–10)) compared to VA group (9 (9–9)) (*p *< 0.001) (Table 5). The Wilcoxon test results showed different performance scores in the VSA group immediately and 2 months after the intervention. The performance scores of students in the VR group were significantly different immediately and 2 months after the intervention compared to before the intervention, but no significant difference was observed in the mean score 2 months after the intervention compared to immediately after the intervention (Table 5).

**Table 5:** Comparison of mean performance scores between the VSA and the VR groups before, immediately, and 2 months after the intervention.

Performance score	VSA	VR	*p* value (between‐groups)
Before the intervention			
Mean ± SD	4.18 ± 1.30	3.92 ± 1.29	0.467^a^
95% CI for mean	3.67–4.70	3.41–4.44	
Immediately after the intervention (median (Q1–Q3))	9 (9–9)	9 (8–10)	0.780^b^
Two months after the intervention (median (Q1–Q3))	10 (9–10)	9 (9–9)	0 < 001^b^
Effect Size (Kendall's W) *p* value (within‐groups)	*W* = 0.787 *p* < 0.001^c^	*W* = 0.821 < 0.001^c^	

^a^Independent samples *t*‐test.

^b^Mann–Whitney *U* test.

^c^Friedman test.

## 4 Discussion

This study aimed to compare two educational methods, VSA and VR, on nursing students' BLS competency. According to the study findings, both educational methods significantly improved the knowledge, attitude, and performance of students, which is consistent with previous studies [12, 19, 23, 24, 25].

While both methods yielded positive outcomes, the VSA method demonstrated superior effectiveness in knowledge retention and long‐term performance stability. This advantage may stem from the extended engagement with recorded videos, allowing students to observe their performance repeatedly. This continuous visual feedback, combined with immediate instructor input, facilitated error recognition, correction, and ultimately, enhanced knowledge and practical skills over time. It is important to note, however, that while the statistical difference in score improvement was significant, the clinical significance of this margin requires further investigation, as the controlled educational environment differs markedly from the high‐stress clinical setting. The VSA method effectively stabilized existing BLS knowledge and facilitated the acquisition of new information. Furthermore, reviewing performance videos allowed for targeted identification and correction of CPR weaknesses, leading to improved and sustained long‐term performance. Notably, the VSA method fostered a positive attitude towards BLS, a crucial element for prompt and accurate application in emergencies. This positive shift stemmed from increased self‐confidence, a high sense of responsibility, and enhanced motivation in learning BLS [12, 26, 27]. The continuous self‐observation and reaction inherent in this method cultivated a positive learning experience, boosting both motivation and confidence in individual BLS abilities. This contrasts with Szöllősi et al.'s findings, which indicated limited impact of video on CPR performance [28]; this discrepancy may be attributed to differences in the feedback mechanism or the duration of the intervention in their study.

The results showed that VR improved students' knowledge, attitude, and performance related to CPR, which is consistent with previous similar studies [29, 30, 31, 32, 33]. Several studies have shown that the use of VR technology in medical education can lead to improved knowledge, attitude, and performance of learners. For example, Sung et al. [34] in a systematic review and meta‐analysis concluded that VR is an effective educational tool in healthcare education and can enhance learning. Simulating emergencies in VR enhances the sense of presence and a more accurate understanding of the importance of CPR in real‐life situations. This method improves students' attitudes towards CPR skills by creating a sense of engagement and interaction. Additionally, VR environments reduce stress in simulated situations by providing a safe space for repeated practice without the immediate pressure of a live patient [35].

Recent high‐quality randomized controlled trials (RCTs) provide nuanced perspectives on VR's efficacy that align with our findings. Leszczyński et al. conducted an RCT on BLS training and found that while VR significantly improved psychomotor skills and confidence compared to traditional training, it did not necessarily outperform other active learning methods in all competency domains [36]. Similarly, Issleib et al. reported in a non‐inferiority trial that VR was a viable alternative for medical staff on general wards, achieving comparable outcomes to standard training in terms of basic life support adherence, though they noted that VR's primary value lies in accessibility and standardized scenario repetition rather than superior skill acquisition [37]. These studies support our observation that VR is an effective tool for initial skill acquisition and confidence building, but may not inherently guarantee superior long‐term retention compared to methods that emphasize reflective practice, such as VSA.

However, the current study did not find evidence to support the long‐term superiority of VR over VSA in knowledge retention or sustained performance. The VR group's performance gains appeared to plateau compared to the continuous improvement seen in the VSA group. This finding suggests that the novelty effect of VR may diminish over time without complementary traditional practice. Furthermore, technical challenges encountered during the VR intervention, such as heavy headsets and ill‐fitting hand controls, likely influenced students' learning and performance [28, 32]. This aligns with Wiltvank et al.'s findings, which indicated that despite the potential of VR in BLS training, technical complexities, the requirement for in‐person support, and software limitations can diminish its overall effectiveness [22]. Additionally, Leszczyński et al. highlighted that while immersive technologies are valuable, the physical realism of training tools (such as using cadavers or high‐fidelity manikins) remains critical for developing tactile feedback in procedures like tourniquet application or chest compressions [38]. This suggests that VR's lack of physical resistance, compared to tactile feedback from manikins or video‐based self‐correction, might explain why VSA offered better long‐term stabilization of psychomotor skills in our study.

## 5 Conclusion

This study demonstrates that while both VSA and VR are effective in enhancing students' knowledge, attitudes, and performance in BLS, VSA is superior in stabilizing knowledge and improving long‐term performance. Consequently, integrating these methods with clinical training can optimize emergency care quality, leveraging VSA for self‐regulated learning and VR for immersive situational awareness. To address the limitations of this pilot investigation, particularly its small sample size and exclusive focus on nursing students, future research should prioritize large‐scale, multi‐center randomized controlled trials to validate these findings across diverse healthcare populations and assess the sustained transfer of skills to high‐stress clinical environments.

## 6 Limitations and Future Implications

This study has several limitations that should be considered when interpreting the findings. First and foremost, this study should be regarded as a pilot investigation due to its small sample size. The limited number of participants restricts the statistical power of the analysis and may affect the generalizability of the results. Therefore, these findings should be interpreted with caution, and larger, multi‐center randomized controlled trials are necessary to confirm the efficacy of these educational interventions. Second, the sample consisted exclusively of nursing students, who represent a novice group. Consequently, the results may not be generalizable to other populations, such as experienced nurses, other healthcare professionals, or laypersons, as their learning patterns and baseline competencies may differ significantly. Third, the use of a researcher‐made questionnaire presented moderate reliability, which may have introduced measurement error compared to standardized instruments. Additionally, potential confounding from instructor feedback in both groups could have influenced student performance, as consistent guidance might have masked differences between the VSA and VR methods. Fourth, the VSA method relies heavily on students' ability to accurately self‐evaluate their performance. This approach is inherently subjective and susceptible to biases such as the Dunning–Kruger effect (over‐ or under‐confidence), which could lead to inaccurate measurements of true competency. Fifth, the effectiveness of VR training is contingent upon the quality and realism of the simulation technology used; variations in hardware or software fidelity could influence outcomes. Finally, performance assessed in a controlled simulated environment may not fully predict performance in high‐stress, unpredictable clinical settings, where time pressure and emotional stress play critical roles.

## Author Contributions

**Mohsen Hamidian:** conceptualization, investigation, writing – original draft, writing – review and editing, methodology, data curation, supervision, software. **Hamid Salehiniya:** conceptualization, validation, methodology, formal analysis, project administration, supervision, data curation, software. **Anis Jor:** data curation, project administration, methodology, writing – review and editing, writing – original draft, conceptualization. **Hakimeh Sabeghi:** conceptualization, investigation, writing – original draft, writing – review and editing, methodology, data curation, project administration, supervision, resources, visualization, validation.

## Funding

The authors have nothing to report.

## Ethics Statement

The study was conducted in accordance with the Declaration of Helsinki, and was approved by the Ethics Committee of Birjand University of Medical Sciences (IR.BUMS.REC.1403.191).

## Consent

The informed consent of nursing students was assumed upon participation.

## Conflicts of Interest

The authors declare no conflicts of interest.

## Transparency Statement

The corresponding author, Hakimeh Sabeghi, affirms that this manuscript is an honest, accurate, and transparent account of the study being reported; that no important aspects of the study have been omitted.

## Data Availability

Data are available upon reasonable request.
